# Inhibition of Mitochondrial Complex III Causes Dopaminergic Neurodegeneration by Redox Stress in *Caenorhabditis elegans*

**DOI:** 10.1101/2025.10.21.683798

**Published:** 2025-10-23

**Authors:** Javier Huayta, Joel N. Meyer

**Affiliations:** 1 Nicholas School of the Environment, Duke University, Durham NC, USA.

## Abstract

Environmental factors including chemical exposures are important contributors to Parkinson’s disease (PD). Nearly all well-validated chemicals involved in PD affect mitochondria, and the great majority of those identified inhibit mitochondrial complex I, causing ATP depletion and oxidative stress. We hypothesized that inhibition of mitochondrial complex III would also cause dopaminergic neurodegeneration. Using *Caenorhabditis elegans* to evaluate the *in vivo* effects of the complex III-inhibiting pesticides antimycin A and pyraclostrobin, we found that both caused neurodegeneration. Neurodegeneration was specific to the dopamine neurons, and complex III inhibition caused a more-oxidized cellular environment in those neurons. Pharmacological and genetic antioxidant interventions rescued neurodegeneration, but energetic rescue attempts did not. Finally, optogenetic production of superoxide anion specifically at complex III caused dopaminergic neurodegeneration. Thus, redox stress at complex III is sufficient for dopaminergic neurodegeneration, and redox stress following chemical inhibition is necessary for dopaminergic neurodegeneration *in vivo* in *C. elegans*.

## Introduction

Parkinson’s disease (PD), characterized by loss of dopaminergic neurons in the *substantia nigra*, affected 10 million people globally in 2021 (1) and is projected to increase to 25.2 million by 2050 (2). This increase is an outcome of an increasingly older population worldwide, particularly in the most polluted parts of the world (3). Environmental factors are important contributors to PD, and many studies have demonstrated the role of chemical exposures (4, 5). Most if not all of the chemicals linked to onset of PD also affect mitochondria (6, 7). However, there is strong evidence for association with PD for only a few chemicals, and because relatively few people are exposed to significant amounts of those chemicals, they collectively likely explain only a small fraction of idiopathic PD. It is not feasible to comprehensively test all the chemicals that induce mitochondrial dysfunction for involvement in PD or parkinsonism, as these number in the hundreds to thousands (8). However, mitochondrial dysfunction is a broad term that includes many specific mechanisms of toxicity (9–11). These include inhibition of all four electron transport chain complexes, ATP synthase, and Krebs cycle enzymes; redox cycling; mitochondrial DNA damage; and uncoupling of ATP production from oxygen consumption. Therefore, to prioritize chemicals for testing, it would be valuable to identify which specific types of mitochondrial toxicity drive dopaminergic neurodegeneration. To date, there is limited evidence for the role of specific types of mitochondrial dysfunction in dopaminergic neurodegeneration. There is strong evidence for complex I inhibition (12, 13), and one report that mitochondrial uncoupling may be protective against dopaminergic neurodegeneration (14). A study using cell culture models examined 21 different pesticides that inhibited complex I, II, or III, finding evidence that some inhibitors of complex I and complex III, but no complex II inhibitors, were specifically toxic to dopaminergic cells in culture (15).

One reason to hypothesize that not all forms of mitochondrial dysfunction cause dopaminergic neurodegeneration is that these forms of mitochondrial dysfunction do not all cause the same downstream molecular events. Two common downstream outcomes of mitochondrial toxicity are redox imbalance and ATP depletion (16, 17), but these key events will not universally result from all forms of mitochondrial toxicity. For example, complex I inhibitors will typically both decrease ATP availability and increase reactive oxygen species (ROS) by diverting electron flow from ubiquinone to molecular oxygen (12), while a mitochondrial uncoupler may also decrease ATP levels but decrease ROS by decreasing mitochondrial membrane potential (MMP) (14). There is some literature addressing the importance of redox imbalance in comparison to ATP depletion in mitochondrial toxicant-induced dopaminergic neurodegeneration. For example, two studies indicate that although the complex I inhibitor rotenone both decreased ATP levels and increased ROS, it was ROS that drove neurodegeneration (12, 18). However, careful mechanistic analysis of this sort after chemical exposure has been rare. It is important to determine which forms of mitochondrial dysfunction are important for both regulatory and mechanistic reasons.

In this work, we use the model organism *C. elegans*, a well-characterized model for neurotoxicity (19), to evaluate the *in vivo* effects of complex III inhibition on dopaminergic neurodegeneration, ATP levels, and redox state. To ensure that results are physiologically relevant and specific to dopaminergic neurodegeneration, we use exposure concentrations that cause mild and no organismal-level toxicity, and compare outcomes in dopaminergic neurons to several other neuron types present in *C. elegans*. We also evaluate potential exacerbation of dopaminergic neurodegeneration by presence of α-synuclein. We demonstrate that developmental exposure *in vivo* to pesticides antimycin A and pyraclostrobin leads to dopaminergic neurodegeneration. Finally, using a variety of pharmacological, genetic, and optogenetic tools, we show that neurodegeneration is caused by increased mitochondrial ROS at complex III.

## Results

### Pesticides that inhibit mitochondrial complex III cause specific dopaminergic neurodegeneration *in vivo*, in the absence of obvious generalized organismal toxicity

We first asked whether exposure to complex III inhibitors would cause specific dopaminergic neurodegeneration. We took two steps to ensure that we were measuring a specific outcome: 1) we identified concentrations that caused relatively mild organismal-level toxicity (decreased larval growth), and 2) we compared effects in dopaminergic neurons to all other populations of neurons.

**Fig. 1.**
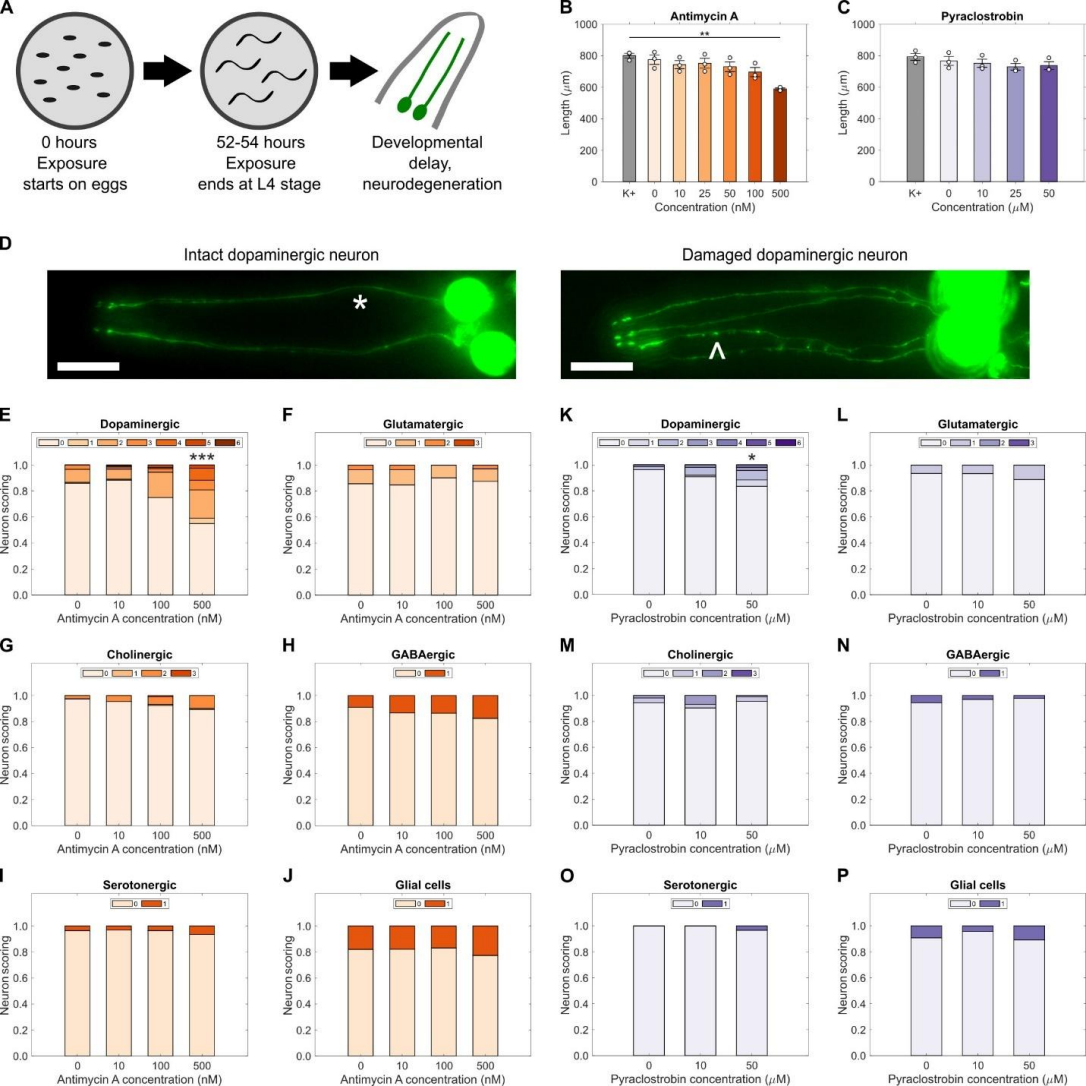
Mitochondrial complex III inhibition causes specific dopaminergic neurodegeneration. (**A**) Chemical developmental exposure occurs from the embryo to the L4 stage, after 52–54 hours endpoint measurement is performed. Length of worms exposed to increasing concentrations of (**B**) antimycin A and (**C**) pyraclostrobin during development. *N* = 3 biological replicates, *n* = 50 – 100 worms per treatment per replicate, error bars SEM, one-way ANOVA with Dunnet’s post-hoc test, **P < 0.01. (**D**) Representative images of intact (*) and damaged neurites (^) of dopamine neurons. Neuronal damage scoring distribution for (**E**) dopaminergic, (**F**) glutamatergic, (**G**) cholinergic, (**H**) GABAergic, (**I**) serotonergic, and (**J**) glial cells located in the head of worms developmentally exposed to antimycin A. Neuronal damage scoring distribution for (**K**) dopaminergic, (**L**) glutamatergic, (**M**) cholinergic, (**N**) GABAergic, (**O**) serotonergic, and (**P**) glial cells located in the head of worms developmentally exposed to pyraclostrobin. *N* = 3 biological replicates, *n* = 40 dendrites for dopaminergic, glutamatergic, and cholinergic neurons, and glial cells, 30 dendrites for GABAergic neurons, 20 dendrites for serotonergic neurons, per treatment per replicate, chi-square test with Bonferroni post-hoc test, *P < 0.05, ***P < 0.001.

Exposure to mitochondrial toxicants causes growth inhibition in *C. elegans* (20, 21). We quantified the effects on *C. elegans* growth of developmental exposure to two inhibitors of coenzyme Q – cytochrome *c* oxidoreductase (complex III) of the ETC: antimycin A, a piscicide that binds to the ubiquinone Qi site (20); and pyraclostrobin, a fungicide that binds to the ubiquinone Q0 site (21) (Fig. 1A). We observed a clear decrease in average worm length with increasing antimycin A concentration (Fig. 1B) but could not identify a significant decrease in average worm length after exposure to concentrations of pyraclostrobin up to 50 μM, the limit of solubility (Fig. 1C). We used this data to construct dose response curves (22) and calculated the concentrations causing a reduction of 10% and 25% in average worm length for antimycin A to be 100 and 500 nM respectively (Fig. S1A). We used these EC_10_ and EC_25_ values for subsequent experiments to ensure that our neuronal endpoint measurements were not secondary to generalized whole organism toxicity. Because we could not test pyraclostrobin over 50 μM, we assigned this maximum concentration as our EC_10_ value (Fig. S1B). Therefore, for further experiments, we used these concentrations in addition to low concentrations of 10 nM for antimycin A and 10 μM for pyraclostrobin.

Next, we tested whether these exposures, causing mild or no growth impairment, would cause neurodegeneration. Diminished mitochondrial function is linked to increased neurodegeneration (9), and neurons show strong sensitivity to mitochondrial impairment (10, 11). However, different toxicants generally affect different types of neurons (23). Therefore, we quantified neurodegeneration in multiple neuronal types (defined by neurotransmitter), quantifying morphological irregularities including kinks, blebs, and loss of continuity in dendrites located in the head region (Fig. 1D, S2) using previously described scoring systems (24, 25). Developmental exposure to antimycin A led to a statistically significant increase in dopaminergic neurodegeneration (Fig. 1E). However, we did not find significant degeneration after exposure to antimycin A in glutamatergic (Fig. 1F), cholinergic (Fig. 1G), GABAergic (Fig. 1H), or serotonergic neurons (Fig. 1I), or glial cells (Fig. 1J). Similarly, developmental exposure to pyraclostrobin caused a statistically significant increase in dopaminergic neurodegeneration (Fig. 1K), albeit not as substantial as that caused by 500 nM antimycin A. Comparable to the results with antimycin A, no concentration of pyraclostrobin induced neurodegeneration in other neuron types (Fig. 1L–1O) or glial cells (Fig. 1P).

### Presence of α-synuclein and deletion of mitophagy genes drive neurodegeneration independent of the effects of complex III inhibition

We hypothesized that dopaminergic neurodegeneration induced by inhibition of complex III would be further increased by the presence of other stressors, considering evidence that PD is often the result of a combination of environmental and genetic factors (26). The protein α-synuclein, encoded by the SNCA gene, is implicated in the onset of PD and other human neurodegenerative diseases (26, 27). In these conditions, α-synuclein misfolds and aggregates in Lewy bodies. This α-synuclein buildup can damage neurons and cause symptoms of these neurodegenerative diseases (27). We tested if the presence of α-synuclein would alter the effects of complex III inhibitors. Because *C. elegans* does not produce endogenous α-synuclein, we used a transgenic *C. elegans* strain containing the human SNCA gene localized to dopaminergic neurons (28). We also tested the effect of mutations in *pdr-1* and *pink-1*, the *C. elegans* homologs of the human PRKN and PINK1 Parkinson’s disease genes, respectively. They are involved in mitophagy, and their loss leads to impaired mitochondrial function and PD (29).

**Fig. 2.**
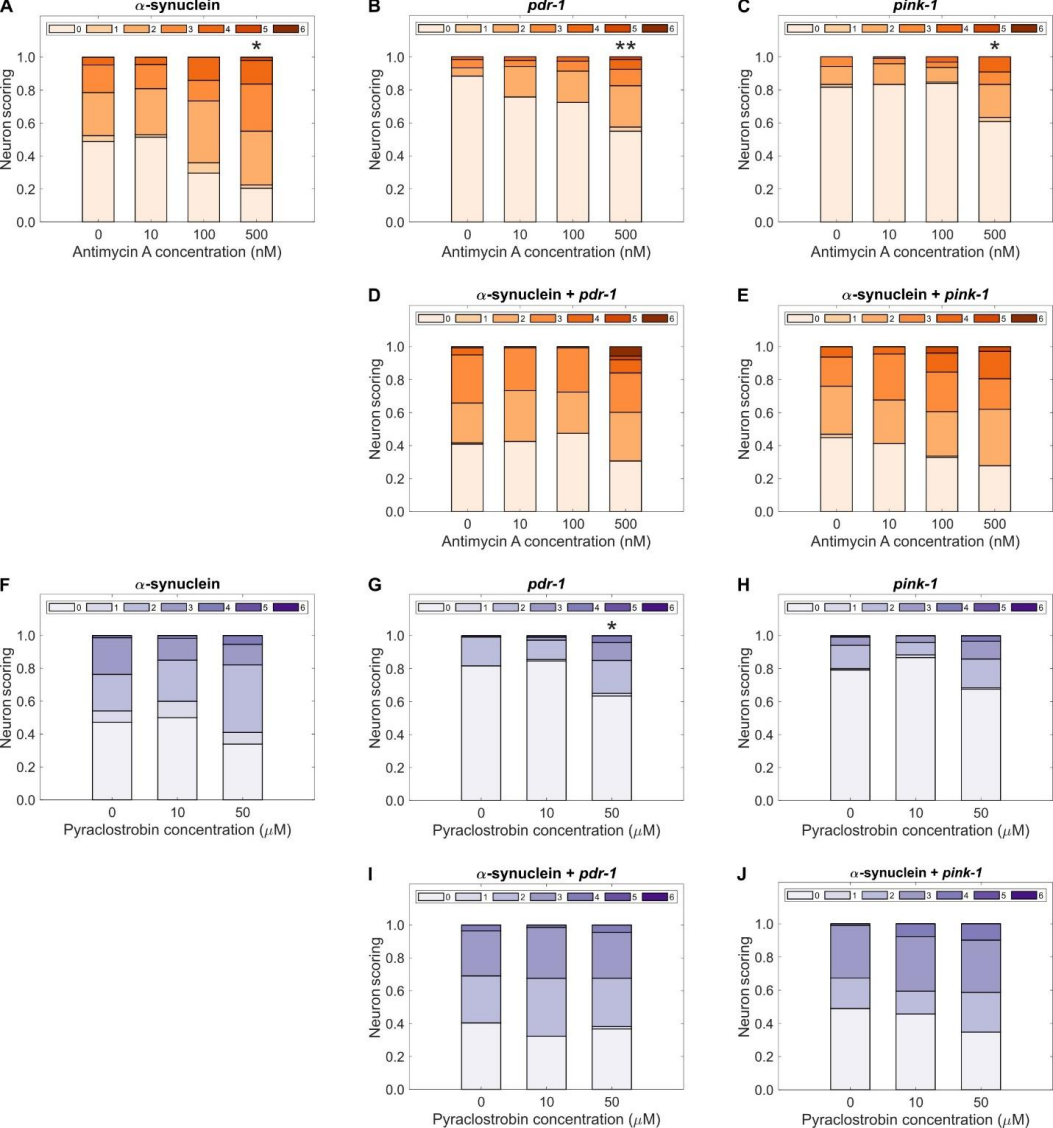
α-synuclein expression and loss of mitophagy genes independently regulate neurodegeneration after developmental inhibition of complex III. Neuronal damage scoring distribution for cephalic dopaminergic neurons in combination with a (**A**) α-synuclein, (**B**) *pdr-1* mutant, (**C**) *pink-1* mutant, (**D**) α-synuclein and *pdr-1* mutant, and (**E**) α-synuclein and *pink-1* mutant backgrounds of worms developmentally exposed to antimycin A. Neuronal damage scoring distribution for cephalic dopaminergic neurons in combination with a (**F**) α-synuclein, (**G**) *pdr-1* mutant, (**H**) *pink-1* mutant, (**I**) α-synuclein and *pdr-1* mutant, and (**J**) α-synuclein and *pink-1* mutant backgrounds of worms developmentally exposed to pyraclostrobin. *N* = 3 biological replicates, *n* = 40 dendrites per treatment per replicate, chi-square test with Bonferroni post-hoc test, *P < 0.05, **P < 0.01.

The expression of α-synuclein alone had a large effect on dopaminergic neurons at all concentrations of antimycin A and pyraclostrobin tested, including controls (Fig. 2). The high concentration of 500 nM antimycin A still had a significant effect on dopaminergic neurodegeneration in the presence of α-synuclein (Fig. 2A), and the *pdr-1* (Fig. 2B) and *pink-1* (Fig. 2C) mutations. No significant differences in neurodegeneration were found when exposing worms with the presence of both α-synuclein and either *pdr-1* (Fig. 2D) or *pink-1* (Fig. 2E) backgrounds to antimycin A. The neurodegenerative effect on dopamine neurons observed with 50 μM pyraclostrobin was recapitulated only for worms with the *pdr-1* background (Fig. 2G), but not for those with the α-synuclein (Fig. 2F) or *pink-1* (Fig. 2H) backgrounds. Exposure to pyraclostrobin in combination with the presence of α-synuclein and either *pdr-1* (Fig. 2I) or *pink-1* (Fig. 2J) mutations did not cause significant differences in neuronal damage. Finally, we considered that developmental inhibition of complex III could sensitize dopaminergic neurons to further chemical insults. To test this, we exposed worms developmentally exposed to antimycin A to secondary challenge with the well-characterized dopaminergic neurotoxin 6-hydroxydopamine (6-OHDA) (30). Worms exposed to antimycin A followed by 6-OHDA challenge show a significant increase in dopaminergic neurodegeneration compared to control worms exposed only to 6-OHDA (Fig. S3).

### Complex III inhibition generates an oxidized redox state in dopaminergic neurons

Inhibition of complex III may decrease the ATP:ADP ratio as electron transfer and proton pumping are inhibited, at least if the exposure is high enough. It may also lead to generation of ROS as electrons leak to oxygen (31, 32). However, there is limited evidence for *in vivo* impacts of complex III inhibition, in particular in specific cells. Furthermore, these effects are dose-dependent, raising the question of whether they occurred in our experimental conditions. Therefore, we characterized how our chemical exposures altered bioenergetic and redox balance. We used 500 nM antimycin A and 50 μM pyraclostrobin because these were the concentrations that induced significant dopaminergic neurodegeneration after developmental exposure. We evaluated the redox state in dopaminergic neurons by using a roGFP reporter under the *dat-1* promoter (33, 34). Similarly, we quantified the ATP:ADP ratios of mitochondria in dopaminergic neurons by using the PercevalHR reporter under the *dat-1* promoter (34, 35).

**Fig. 3.**
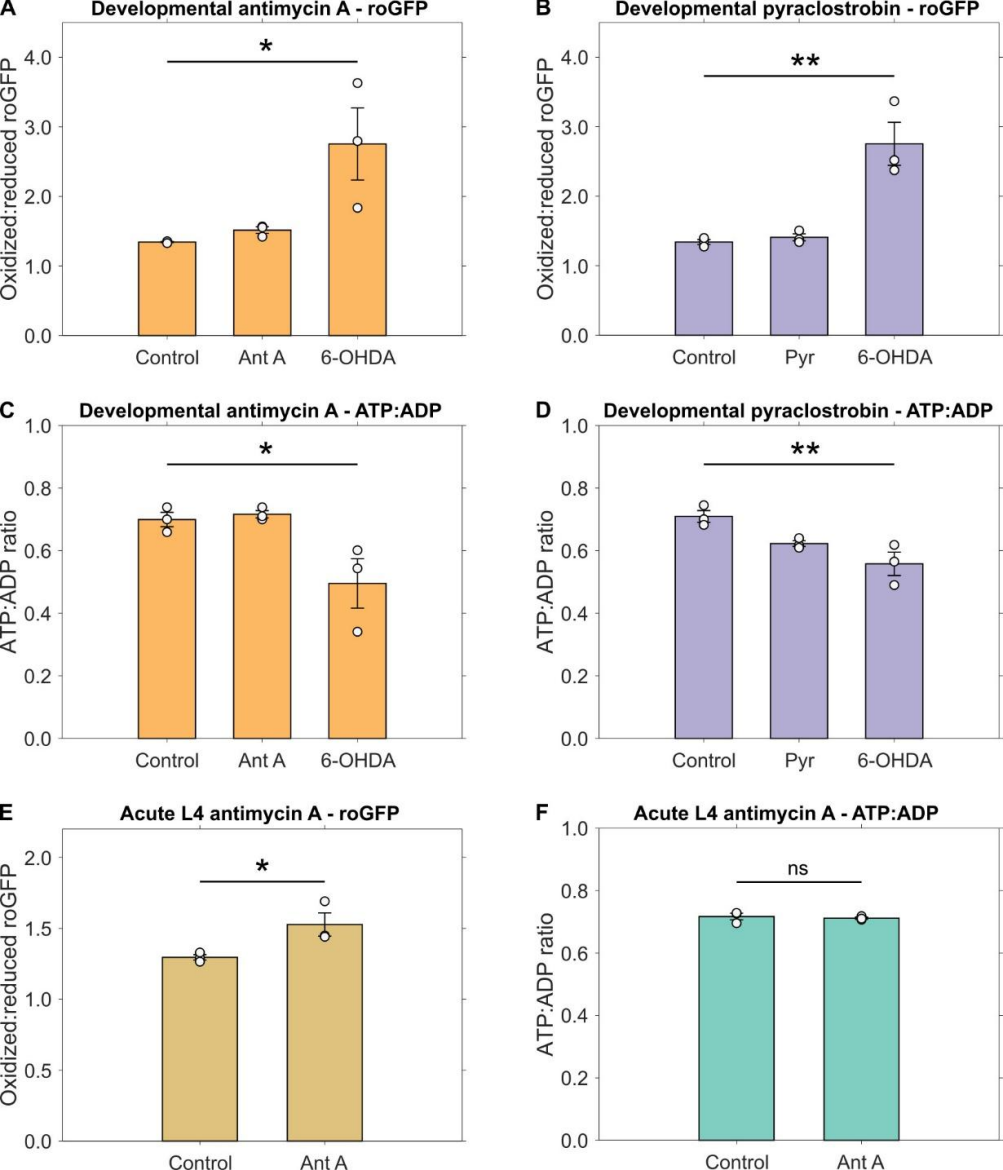
Complex III inhibition alters dopaminergic redox but not bioenergetic state. Oxidized to reduced roGFP ratio in cephalic dopaminergic neurons of *C. elegans* developmentally exposed to (**A**) 500 nM antimycin A or (**B**) 50 μM pyraclostrobin. ATP to ADP ratio as measured using the PercevalHR reporter in cephalic dopaminergic neurons of *C. elegans* developmentally exposed to (**C**) 500 nM antimycin A or (**D**) 50 μM pyraclostrobin. 6-OHDA included as positive control. (**E**) Oxidized to reduced roGFP ratio and (**F**) ATP to ADP ratio as measured using the PercevalHR reporter in cephalic dopaminergic neurons of *C. elegans* exposed to 10 μM antimycin A for 2.5 hours at the L4 larval stage. *N* = 3 biological replicates, *n* = 40 neurons per treatment per replicate, one-way ANOVA test with Dunnett’s post-hoc test, ns *P* > 0.05, *P < 0.05, **P < 0.01.

We found that after developmental inhibition of complex III, neither 500 nM antimycin A (Fig. 3A) nor pyraclostrobin (Fig. 3B) caused an increase in oxidized:reduced roGFP. We used short-term exposure to the dopaminergic neurotoxin 6-OHDA, previously reported to induce an increase in oxidized:reduced roGFP (12, 34), as a positive control. Similarly, neither developmental exposure to 500 nM antimycin A (Fig, 3C) nor 50 μM pyraclostrobin (Fig. 3D) led to significant changes in the ATP:ADP ratio in dopaminergic neurons. We also used 6-OHDA as a positive control for these tests. We considered that the 52 to 54 hours duration of developmental exposure may have enabled the worms to produce compensatory mechanisms to ameliorate mitochondrial ROS generation (36, 37) or energetic challenge (38, 39). To test this, we performed an acute exposure to a high concentration of 10 μM antimycin A (20 times the high concentration for developmental exposure of 500 nM) for 2.5 hours on L4 stage worms. This short exposure led to a significant increase in oxidized:reduced roGFP (Fig. 3E), but no significant change in ATP:ADP ratio was measured (Fig. 3F). Notably, worms affected by this acute exposure to antimycin A did not show significant neurodegeneration immediately after exposure (Fig. S4), indicating that this exposure was too short to enact the neurodegenerative effects of antimycin A.

### S3QEL-2 rescues neurodegeneration caused by complex III inhibition

**Fig. 4.**
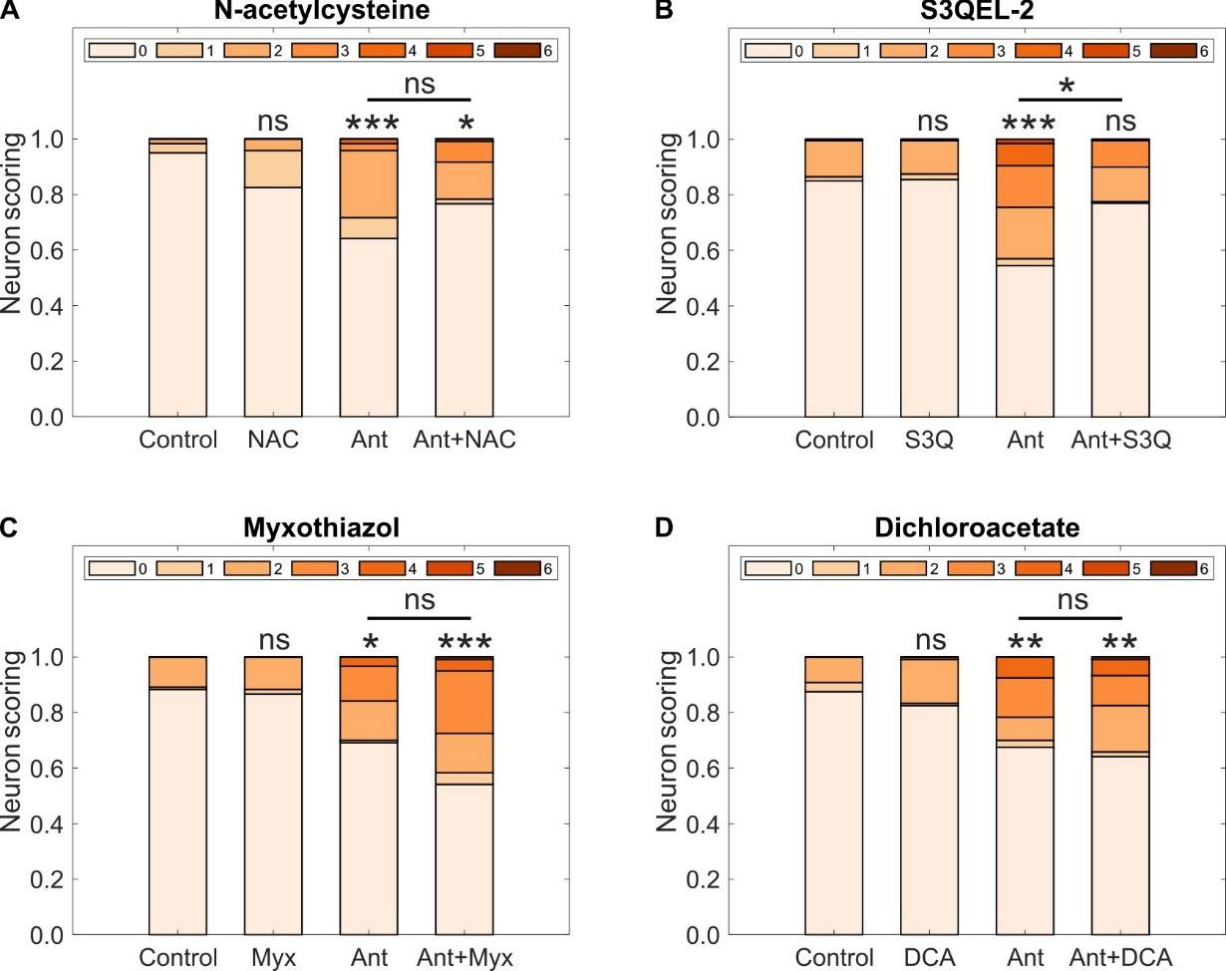
Antioxidant rescue of neurodegeneration after developmental inhibition of complex III. Neuronal damage scoring distribution for cephalic dopaminergic neurons after developmental exposure to 500 nM antimycin A in combination with (**A**) 2.5 mM N-acetylcysteine, (**B**) 100 μM S3QEL-2, (**C**) 10 μM myxothiazol, and (**D**) 25 mM dichloroacetate. *N* = 3 biological replicates for (**A**), (**C**) and (**D**), 4 biological replicates for (**B**), *n* = 40–60 dendrites per treatment per replicate, chi-square test with Bonferroni post-hoc test, ns *P* > 0.05, *P < 0.05, **P < 0.01, ***P < 0.001.

We next directly tested whether neurodegeneration induced by inhibition of complex III is caused by redox or bioenergetic stress by performing pharmacological co-exposures. N-acetylcysteine (NAC) is a well-characterized antioxidant that works by several mechanisms: it is a glutathione precursor; it directly reacts with free radicals through its thiol group; it acts as reducing agent that can break disulfide bonds in inappropriately oxidized proteins; and it promotes the production of intracellular hydrogen sulfide that also serves as an antioxidant (40–42). We developmentally exposed *C. elegans* to 500 nM antimycin A in conjunction with 2.5 mM NAC (Fig. 4A). NAC by itself did not have a significant effect on neurodegeneration, and an apparent decrease in antimycin A-mediated neurodegeneration with NAC co-exposure did not reach statistical significance.

We next tested S3QEL-2, a selective inhibitor of superoxide production from the outer Q-binding site of complex III that acts without affecting oxidative phosphorylation (OXPHOS) (43, 44). Exposure to 100 μM S3QEL-2 alone did not cause detectable neurodegeneration, but rescued antimycin A-mediated neurodegeneration (Fig. 4B). Thus, blocking ROS production at complex III with S3QEL-2 was sufficient to offset the neurodegenerative effects of antimycin A-mediated complex III inhibition.

Myxothiazol has been reported to inhibit the formation and release of semiquinone radicals in complex III (45, 46), indicating a potential opposite effect to antimycin A. However, other reports point towards myxothiazol stimulating the production of hydrogen peroxide and having inhibiting activity in complex III (20, 47). We tested the effects of 10 μM myxothiazol along with 500 nM antimycin A. Myxothiazol itself had no significant effect on dopaminergic neurodegeneration, but the combination of developmental exposure to both myxothiazol and antimycin A was as bad or worse than antimycin A alone (Fig. 4C). If it is in fact worse, it is possible that this is the result of substrate-dependent enhancement of the release of hydrogen superoxide in complex III or a concentration-dependent effect (47). At a minimum, we can confidently conclude that we did not observe any rescue.

Finally, we tested dichloroacetate (DCA), a synthetic organic acid that inhibits pyruvate dehydrogenase kinase, which inhibits pyruvate dehydrogenase (40, 48). DCA thus enhances the activity of pyruvate dehydrogenase, which in turn converts pyruvate into acetyl-CoA that is used for ATP production in mitochondria (49, 50). By diverting pyruvate away from less-energetically favorable glycolysis, DCA treatment may provide a bioenergetic rescue. Developmental exposure to 25 mM dichloroacetate did not cause any significant increase in dopaminergic neurodegeneration and did not alter the effect of antimycin A on dopaminergic neurons (Fig. 4D).

We also evaluated the effects of all four chemicals on dopaminergic redox and bioenergetics states. Developmental exposure to 2.5 mM NAC, 10 μM myxothiazol, and 25 mM dichloroacetate did not cause significant changes in oxidized:reduced roGFP, either by themselves or in combination with antimycin A (Fig. S5). Developmental exposure to 500 nM antimycin A caused a significant increase in oxidized:reduced roGFP for S3QEL-2, but 100μM S3QEL-2 did not, and rescued 500 nM antimycin A-induced oxidation (Fig. S5). Conversely, neither developmental exposure to 2.5 NAC, 100 μM S3QEL-2, 10 μM myxothiazol, 25 mM DCA, 500 nM antimycin A, nor their combinations produced significant changes in ATP:ADP ratios in dopaminergic neurons (Fig. S6). These results, in conjunction with our description of rescue of dopaminergic neurodegeneration mediated by the complex III-specific inhibitor of ROS production S3QEL-2, are consistent with dopaminergic neurodegeneration from complex III inhibition being caused by ROS production at this site.

### Mitochondrial superoxide anion regulates neurodegeneration caused by complex III inhibition

**Fig. 5.**
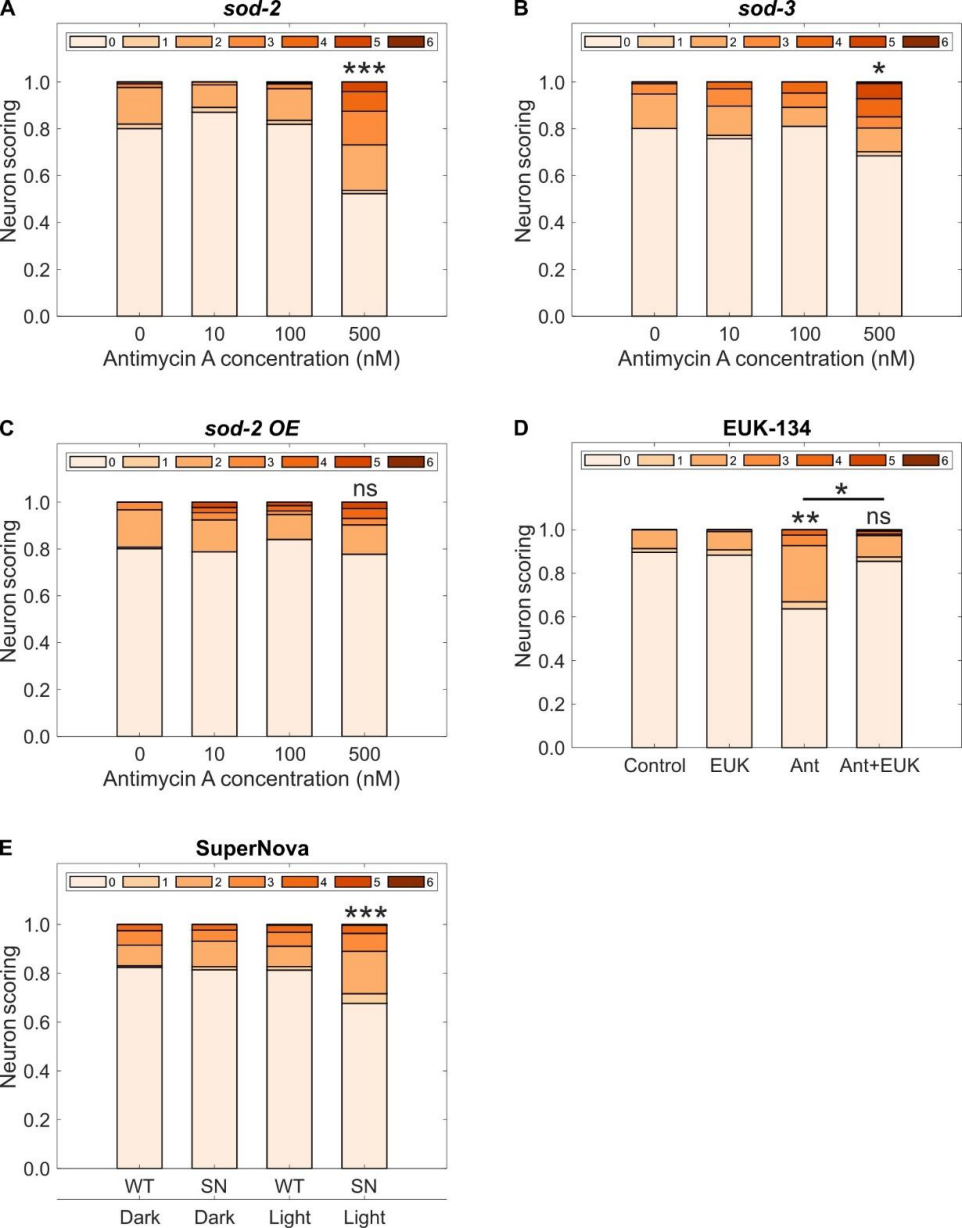
Mitochondrial superoxide anion regulates neurodegeneration linked to complex III inhibition. Neuronal damage scoring distribution for cephalic dopaminergic neurons after developmental exposure to antimycin A in a (**A**) *sod-2* mutant background, (**B**) *sod-3* mutant background, and a (**C**) *sod-2* over expressing strain. (**D**) Neuronal damage scoring distribution after developmental exposure to 500 nM antimycin A in combination with 0.5 mM EUK-134. (**E**) Neuronal damage scoring distribution after optogenetic activation of complex III SuperNova during development. *N* = 3 biological replicates for (**B**), (**C**), and (**D**), 5 biological replicates for (**A**), and (**E**), *n* = 50–80 dendrites per treatment per replicate, chi-square test with Bonferroni post-hoc test, ns *P* > 0.05, *P < 0.05, **P < 0.01, ***P < 0.001.

S3QEL-2 is described as a specific inhibitor of superoxide anion production in complex III (43), but most characterization of S3QEL-2 effects has been *in vitro*. To validate our S3QEL-2-based mechanistic evidence for a causative role for superoxide anion production, we manipulated mitochondrial manganese superoxide dismutase, which has a primary role in scavenging superoxide anion radicals in mitochondria (51). *C. elegans* carries two mitochondrial superoxide dismutase genes, *sod-2* and *sod-3* (52). Loss-of-function mutations in *sod-2* or *sod-3* did not dramatically increase dopaminergic neurodegeneration on their own or after antimycin A exposure (Fig. 5A–B). However, it is possible that complementary mechanisms that target mitochondrial ROS are sufficient to ameliorate its effects (36). With this consideration, we evaluated if increasing superoxide dismutase availability would influence neurodegeneration. Using a strain containing both the dopaminergic reporter and multiple copies of *sod-2* causing overexpression (53), we tested for the effects of complex III inhibition. Increasing expression of *sod-2* abrogated the neurodegenerative effects of exposure to 500 nM antimycin A (Fig. 5C). As a second test of whether supplementation of superoxide dismutase activity reduces dopaminergic neurodegeneration, we performed chemical rescue with the superoxide dismutase and catalase mimetic EUK-134 (54). This synthetic antioxidant converts superoxide anion into hydrogen peroxide and then converts hydrogen peroxide into water and oxygen (55). We found that the addition of 0.5 mM EUK-134 during development to worms exposed to 500 nM antimycin A significantly reduced the dopaminergic neurodegeneration caused by antimycin A itself (Fig. 5D).

With three lines of evidence that complex III inhibitors cause dopaminergic neurodegeneration via production of superoxide anion, we finally asked whether artificial production of superoxide anion specifically at complex III would be sufficient to cause dopaminergic neurodegeneration. We tested *C. elegans* containing the dopaminergic reporter and SuperNova appended to *ucr-2.3* (56), a gene encoding a complex III core protein located in the mitochondrial matrix (57). SuperNova is an optogenetically activated protein that upon excitation produces ROS, particularly superoxide anion and singlet oxygen (58). This chromophore enables spatially targeted generation of these ROS at complex III, localized to the mitochondrial matrix (56). We found that worms with light-activated SuperNova during development show significant neurodegeneration when compared to controls kept in the dark (Fig. 5E). This confirms, in a manner independent from antimycin A exposure, that ROS generation at the complex III site causes dopaminergic neurodegeneration in our model.

## Discussion

Exposure to chemicals present in the environment as pollutants is an important contributor to the onset of PD and other neurodegenerative diseases (4). Many of these chemicals are also mitochondrial toxicants (7). However, mitochondrial dysfunction can take many different forms (9–11). We focused this work on inhibition of complex III of the mitochondrial ETC by two different chemicals, antimycin A and pyraclostrobin. We demonstrated that developmental exposure to either chemical led to dopaminergic neurodegeneration in the model organism *C. elegans*. We also showed that this is directly linked to production of ROS in the complex III site, especially that of superoxide anion. Furthermore, we recapitulated dopaminergic neurodegeneration independently of chemical exposure by generating ROS in complex III using a SuperNova construct. These results indicate that inhibition of mitochondrial complex III by environmentally relevant chemicals leads to ROS-mediated dopaminergic neurodegeneration in *C. elegans*.

Exposure to environmental pollutants, many or all of them mitochondrial toxicants, has been reported to contribute to PD (4, 5). In addition, there is strong evidence of mitochondrial dysfunction in PD patients (6), dopaminergic neurons being especially sensitive to mitochondrial dysfunction (59), and disruption of mitochondrial complex I causing dopaminergic neurodegeneration *in vivo* in mice (13) and *C. elegans* (12). However, many chemicals affect mitochondria (60, 61), and these exposures can affect multiple aspects of mitochondrial function (62), making it difficult to discern which specific types and mechanisms are more important for the onset of dopaminergic neurodegeneration. Complex III inhibition, like complex I inhibition, can both deplete ATP and cause ROS generation, suggesting that complex III inhibition might, like complex I inhibition, cause dopaminergic neurodegeneration. We focused on inhibition of complex III using the piscicide antimycin A (20) and fungicide pyraclostrobin (21). We found that both chemicals caused dopaminergic-specific neurodegeneration at concentrations low enough to not cause whole-organism toxicity. Neurodegeneration was not observed in other types of neurons. Furthermore, we found that superoxide anion generated in the complex III site is sufficient to cause dopaminergic neurodegeneration in *C. elegans*, and that rescue of superoxide anion production after chemical inhibition of complex III rescued neurodegeneration. A critical role of superoxide anion production in dopaminergic neurodegeneration has also been demonstrated for complex I inhibition (12, 18), raising the question of whether this will be a common feature of all chemicals that cause dopaminergic neurodegeneration via mitochondrial toxicity.

However, there are clearly other factors involved in dopaminergic neurodegeneration, raising the question of how they interact. One such factor is α-synuclein aggregation, which has a large role in PD (27). Interestingly, although overexpression of human SNCA (30) caused very significant dopaminergic neurodegeneration on its own, antimycin A and pyraclostrobin appeared to have roughly additive effects when α-synuclein was present. Although our experimental design does not formally test additivity, both factors have independent effects, alone and together. Similarly, PRKN and PINK1 are mitophagy regulators that are linked to PD epidemiologically and mechanistically, due to their role in removing damaged proteins and mitochondria (6, 29). Once again, we did not find evidence that *pdr-1* and *pink-1* mutations had a synergistic effect on chemically induced dopaminergic neurodegeneration. Interestingly, worms having both α-synuclein production and either a *pdr-1* or *pink-1* mutant background showed a loss of the significant effect of complex III inhibition. A caveat to these results is that it is possible that the high baseline of neuronal damage caused by α-synuclein in our model caused an obscuring effect of any potential additive effect. Notably, α-synuclein is reported to cause mitochondrial fragmentation in *C. elegans*, with PINK1 and parkin coexpression rescuing this inhibition of mitochondrial fusion by α-synuclein (63). This led us to hypothesize a synergistic worsening of the neuronal damage of these combined backgrounds, but our results do not support this. We previously reported that *pdr-1* sensitizes *C. elegans* to dopaminergic neurodegeneration driven by 6-OHDA exposure, and *pink-1* had a protective effect under the same conditions (64). This coincides with our observations of dopaminergic neurodegeneration for both backgrounds, with *pink-1* worms having less deviation from the control than those with a *pdr-1* mutation.

PD is the second most common neurodegenerative disease and the most common movement disorder, affecting approximately 10 million people worldwide globally as of 2021 (1). This is likely to increase with a growing population of older people. Therefore, understanding the causes of PD to prevent cases is of critical importance. Exposure to environmental pollutants such as pesticides and heavy metals, have been linked to the onset of PD (5, 65, 66), but epidemiological evidence is scarce and causality often unclear. Mitochondrial toxicity is a mode of action for these toxicants, with strong evidence for complex I inhibition as causative of parkinsonism and dopaminergic neurodegeneration (12, 13, 38). A recent screening of various pesticides targeting complexes I, II, and III using human cell culture models, found evidence that inhibition of both complex I and III led to dopaminergic neurotoxicity (15). We are not aware of other studies testing specific dopaminergic neuron sensitivity to complex III inhibition. Our work provides robust confirmation that 1) complex III inhibition causes dopaminergic neurodegeneration *in vivo*, and 2) that this is directly related to generation of mitochondrial ROS in complex III, providing mechanistic insights on this specific mode of mitochondrial dysfunction. This is important from a regulatory perspective because complex III inhibitors are common in nature, frequently used as pesticides, and increasingly pursued for drug development (67). Additional work in longer-lived models and epidemiological studies focusing specifically on complex III inhibitors will be important to assess the importance of complex III inhibition to PD or Parkinsonism in people. From a mechanistic perspective, the knowledge that mitochondrial ROS, in particular superoxide anion, is at the center of dopaminergic dysfunction may be helpful in the development of preventive and therapeutic approaches.

## Materials and Methods

### *C. elegans* maintenance

*C. elegans* strains. Strains BY200 (*vtIs1*[p*dat-1*::GFP]), DA1240 (*adIs1240*[*eat-4*::sGFP+*lin-15*(+)]), LX929 (*vsIs48*[*unc-17*::GFP]), CZ1632 (*juIs76*[*unc-25*p::GFP+*lin-15*(+)]), GR1366 (*mgIs42*[*tph-1*::GFP+*rol-6*(su1006)]), VT1485 (*maIs188*[*mir-228*p::GFP+*unc-119*(+)], ERS1 (*eraIs1*[*dat-1p*::mCherry, *dat-1p*::hSNCA::Venus]), ERS44 (*eraIs1*[*dat-1p*::mCherry, *dat-1p*::hSNCA::Venus]; *pdr-1*(gk448)), ERS49 (*eraIs1*[*dat-1p*::mCherry, *dat-1p*::hSNCA::Venus]; *pink-1(tm1779)*), UA226 (*pink-1*(tm1779); *vtIs1*[p*dat-1*::GFP]), UA227 (*pdr-1*(tm598); *vtIs1*[p*dat-1*::GFP]), PHX2867 (p*dat-1*::MLS::roGFP), PHX2923 (p*dat-1*::PercevalHR), GA184 (*sod-2*(gk257) I), GA186 (*sod-3*(tm760) X), GA805 (*wuIs156*[*sod-2*(genomic) + *rol-6*(su1006)], and APW125 (*jbm21*[*ucr2.3*::link::SuperNova] III) were maintained at 20 °C on K-agar plates seeded with OP50 *E. coli* (68). Worms were fed OP50 *E. coli* in experiments requiring culture on solid medium because of the limited bacterial lawn obtained with OP50, allowing easier observation of worms (69). HB101 *E. coli* was used in experiments requiring liquid culture because it is less prone to forming bacterial clumps, making it easier to eat for the worms (70). Strain BY200 was a gift from Michael Aschner; strain APW125 was a gift from Andrew Wojtovich; strains ERS1, ERS44, and ERS49 were a gift from Roman Vozdek; and other strains were obtained from the Caenorhabditis Genetics Center (Table S1).

Generation of crossed strains. BY200 male worms were generated by exposing L4 stage worms to 33 °C for 4 hours. After 2–3 days, the offspring were screened for males and transferred to new plates containing one hermaphrodite and three males each. This step was necessary to increase the number of available males in the next generation. After 2–3 days, new plates were prepared containing one hermaphrodite of either of the GA184, GA185, GA805 or APW125 strains and three BY200 second-generation males. After 2–3 days, putative homozygous crosses were first verified for the visible BY200 dopaminergic neuron fluorescence. Putative homozygous crosses with strain GA805 were further screened for the *rol* phenotype. Putative homozygous crosses with strains GA184, GA185, and APW125 were verified by PCR genotyping (Table S2).

Age-synchronization by bleaching treatment. For experiments requiring age-synchronization, a non-starved population of day 1–2 adults was collected in a 15 mL tube from K-agar plates by washing with K-medium (68). Worms were allowed to settle for 2–3 minutes, and the supernatant discarded. Worms were then treated with 5 mL of K-medium containing final concentrations of 0.4 N sodium hydroxide and 20% v/v sodium hypochlorite for eight minutes. The bleaching reaction was quenched by raising the volume to 15 mL with K-medium, centrifuged at 2200 RCF for 2 minutes, and the supernatant discarded. The last step was repeated two more times to recover the embryos for exposure experiments. We tested whether this bleaching treatment would affect dopaminergic neurodegeneration after the worms reached the L4 larval stage. We found no significant effect on dopaminergic neurodegeneration by this treatment compared to worms that were age-synchronized by adult egg-laying (eggs laid for one hour, and then adults removed) or by egg isolation via bleaching followed by L1 synchronization by overnight hatch in liquid without food (Fig. S7).

### Chemical exposure design

Developmental exposure to chemicals. Embryos generated by bleaching treatment were counted on a stereo microscope by transferring 10 μL of K-medium containing embryos to a glass slide. K-medium was added or subtracted until reaching a concentration of 10 embryos/μL. Approximately 100 embryos in 10 μL of K-medium were transferred to each well of a 24-well plate and the volume was increased to a total of 500 μL per well of complete K-medium (0.5 mL of 10 mg/mL cholesterol, 3 mL of 1 M calcium chloride, 3 mL of 1 M magnesium sulfate per 1 L K-medium) containing HB101 *E. coli* bacterial food at a final concentration of OD 2.00, and either 0, 10, 100 or 500 nM of antimycin A (Sigma-Aldrich) or 0, 10, or 50 μM of pyraclostrobin (Sigma-Aldrich) final concentrations. All wells also contained a final concentration of 1% v/v DMSO as vehicle. The 24-well plate was put on a shaker at 20 °C for 52–54 hours to allow worms to reach the L4 larval stage. Afterward, liquid and worms from each well were collected in separate 15 mL tubes, the total volume raised to 10 mL with K-medium, and the worms were allowed to settle. After 2–3 minutes, the supernatant was discarded, and this washing step was repeated two more times. At this point worms were ready for endpoint measurement.

Determination of experimental concentrations. Embryos were treated as described in the preceding paragraph, but with additional concentrations tested during development; 0, 10, 25, 50, 100, or 500 nM antimycin A, or 0, 10, 25, or 50 μM pyraclostrobin final concentrations. After 52–54 hours worms were collected and transferred to unseeded K-agar plates, allowing the worms to disperse on the plate for 5 minutes. Plates containing worms were mounted and imaged using a Keyence BZ-X710 microscope using a Nikon 4X objective. Images were analyzed using the WormSizer add-in in ImageJ following their published protocol to determine worm length of a minimum of 50 individuals per treatment (71). These length values were then used to construct a dose response curve using AAT Bioquest EC_50_ Calculator (22) to determine EC_10_ and EC_25_ concentrations.

6-hydroxydopamine exposure. Worms that were exposed to 6-OHDA as a secondary challenge or as a positive control for roGFP and PercevalHR assays were collected and washed as described in the preceding paragraphs. These worms were then transferred to 24-well plates and well volumes were raised to a total of 500 μL per well of complete K-medium containing 50 μM of 6-hydroxydopamine (Sigma-Aldrich) and 10 μM of L-ascorbic acid (Sigma-Aldrich) final concentrations. Control wells contained 500 μL per well of complete K-medium with 10 μM of L-ascorbic acid. Worms were exposed for one hour and then collected in 15 mL tubes and washed three times as described above. Microscopy was performed immediately after.

Acute antimycin A exposure. Embryos generated by bleaching treatment were counted on a stereo microscope by transferring 10 μL of K-medium containing embryos to a glass slide. Approximately 100 embryos in K-medium were transferred to K-agar plates and allowed to reach the L4 larval stage. After 52–54 hours, worms were collected and transferred to 24-well plates and volumes raised to a total of 500 μL per well of complete K-medium containing 0, or 10 μM of antimycin A final concentrations for 2.5 hours. All wells also contained a final concentration of 1% v/v DMSO as vehicle. Worms were collected in 15 mL tubes after 2.5 hours, and washed as described above. Microscopy was performed immediately after.

### Quantification of neurodegeneration

Microscopy imaging. L4 larval stage worms that previously went through chemical exposure were transferred to 5 μL of 100 mM sodium azide solution on a 2% agarose pad on top of a glass slide. Worms were paralyzed by the sodium azide after one minute and covered with a coverslip. The glass slide was mounted on a Keyence BZ-X710 microscope equipped with a Keyence BZ-X700E metal halide light-source. Z-stacks of individual worms’ heads were acquired using a Nikon 40X objective and a Chroma GFP filter cube with 100 milliseconds of exposure, microscope objective’s pitch of 0.5 μm, and 3×3 binning.

Neuronal damage quantification. Maximum projections of the z-stacks based on maximum intensity were generated, and each dendrite of the cephalic neurons was scored as previously described (24, 25). For dopaminergic neurons: 0 – no damage, 1 – irregular (curves), 2 – less than 5 blebs, 3 – 5 to 10 blebs, 4 – more than 10 blebs and/or breaks, 5 – breaks, 25 to 75% dendrite loss, and 6 – breaks, more than 75% dendrite loss. For cholinergic neurons: 0 – no damage, 1 – irregular (curves), 2 – 1 to 10 blebs, and 3 – more than 10 blebs and/or presence of breaks. For glutamatergic neurons: 0 – no damage, 1 – 1 to 5 blebs, 2 – 6 to 10 blebs, and 3 – more than 10 blebs and/or presence of breaks. For serotonergic neurons, GABAergic neurons and glial cells: 0 – no damage, and 1 – any type of damage.

### Quantification of glutathione redox tone and ATP:ADP ratio in dopaminergic neurons

Worms of strains PHX2867 and PHX2923, expressing mitochondrial-localized roGFP and PercevalHR respectively under the *dat-1* promoter (34), were used to quantify the ATP:ADP ratio and glutathione redox tone in dopaminergic neurons. Worms that previously went through chemical exposure were transferred to 5 μL of K-medium on an 8% agarose pad on top of a glass slide and covered with a coverslip. No paralytic chemicals were used because many interfere with these parameters (72). The glass slide was mounted on a Keyence BZ-X710 microscope equipped with a Keyence BZ-X700E metal halide light-source. Two images per head of an individual worm, focused on the cell bodies of the cephalic dopaminergic neurons, were acquired using a Nikon 40X objective, 25 milliseconds of exposure, and 3×3 binning. The first image was acquired using a 470ex/520em filter, and the second image with a 405ex/520em filter. A custom-made MATLAB script was used to perform feature-based segmentation of the cell bodies in each image, background subtraction, and to determine their mean intensity values. The script then calculated the mean intensity ratios of 405/470 excitation for mitochondrial roGFP as oxidized to reduced glutathione, and the mean intensity ratios of 470/405 excitation for PercevalHR as ATP to ADP ratio.

### Chemical rescue experiments

Embryos generated by bleaching treatment were counted on a stereo microscope by transferring 10 μL of K-medium containing embryos to a glass slide. K-medium was added or subtracted until reaching a concentration of 10 embryos/μL. Approximately 100 embryos in 10 μL of K-medium were transferred to each well of a 24-well plate and raised to a total volume of 500 μL per well of complete K-medium containing HB101 *E. coli* bacterial food at a final concentration of OD 2.00. For each replicate, one well contained no additional chemicals to serve as control, one well contained only the rescue chemical, one well contained 500 nM antimycin A, and one well contained 500 nM antimycin A and the rescue chemical. The rescue chemicals final concentrations were 2.5 mM N-acetylcysteine (Sigma-Aldrich), 100 μM S3QEL-2 (Sigma-Aldrich), 10 μM myxothiazol (Sigma-Aldrich), 25 mM dichloroacetate (Sigma-Aldrich), or 0.5 mM EUK-134 (Sigma-Aldrich). All wells also contained a final concentration of 1% v/v DMSO as vehicle. The 24-well plate was put on a shaker at 20 °C for 52–54 hours to allow worms to reach the L4 larval stage. Afterward, liquid and worms from each well were collected in separate 15 mL tubes, raised to 10 mL with K-medium, and the worms were allowed to settle. After 2–3 minutes, the supernatant was discarded, and this washing step repeated two more times. At this point worms were ready for endpoint measurement.

### Optogenetic induction of SuperNova

Embryos generated by bleaching treatment were counted on a stereo microscope by transferring 10 μL of K-medium containing embryos to a glass slide. Approximately 100 embryos in K-medium were transferred to K-agar plates. SuperNova activation was conducted using the AMUZA 590 nm LED array for Multiwell Plates (12, 56). K-agar plates containing embryos were placed approximately 2 cm above the surface of the LED array location to enable air flow and to keep temperature in the incubator at 20 °C. Worms were under continuous light exposure of 0.3 mW/mm^2^ and allowed to reach the L4 larval stage. Dark controls were placed within the same incubator, in a cardboard box with an aluminum foil lid to prevent light penetration. After 52–54 hours, K-agar plates containing worms were retired from the incubator and microscopy was performed.

### Statistical analysis

MATLAB R2024b (MATLAB 24.2) was used for all statistical testing and graph generation. Statistical tests and sample size are described in their corresponding figure legends.

## Supplementary Material

Supplement 1

## Data Availability

All data needed to evaluate the conclusions of this work are available in the main text or the supplementary materials.

## References

[1] Li M., Ye X., Huang Z., Ye L., Chen C., Global burden of Parkinson’s disease from 1990 to 2021: a population-based study. BMJ Open 15, e095610 (2025). 10.1136/bmjopen-2024-095610 PMC12035419 40288800

[2] Su D., Cui Y., He C., Yin P., Bai R., Zhu J., Lam J. S. T., Zhang J., Yan R., Zheng X., Wu J., Zhao D., Wang A., Zhou M., Feng T., Projections for prevalence of Parkinson’s disease and its driving factors in 195 countries and territories to 2050: modelling study of Global Burden of Disease Study 2021. BMJ 388 (2025). 10.1136/bmj-2024-080952 PMC11881235 40044233

[3] Ray Dorsey E., Bloem B. R., Parkinson’s Disease Is Predominantly an Environmental Disease. J Parkinsons Dis 14, 451 (2024). 38217613 10.3233/JPD-230357 PMC11091623

[4] Chen H., Ritz B., The Search for Environmental Causes of Parkinson’s Disease: Moving Forward. J Parkinsons Dis 8, S9–S17 (2018). 30584168 10.3233/JPD-181493 PMC6311360

[5] Klingelhoefer L., Reichmann H., Pathogenesis of Parkinson disease--the gut-brain axis and environmental factors. Nat Rev Neurol 11, 625–36 (2015). 26503923 10.1038/nrneurol.2015.197

[6] Schapira A. H., Mitochondria in the aetiology and pathogenesis of Parkinson’s disease. [Preprint] (2008). 10.1016/S1474-4422(07)70327-7. 18093566

[7] Helley M. P., Pinnell J., Sportelli C., Tieu K., Mitochondria: A Common Target for Genetic Mutations and Environmental Toxicants in Parkinson’s Disease. Front Genet 8, 177 (2017). 29204154 10.3389/fgene.2017.00177 PMC5698285

[8] Te Lin Y., Lin K. H., Huang C. J., Wei A. C., MitoTox: a comprehensive mitochondrial toxicity database. BMC Bioinformatics 22, 369 (2021). 34266386 10.1186/s12859-021-04285-3 PMC8283953

[9] Johri A., Beal M. F., Mitochondrial Dysfunction in Neurodegenerative Diseases. Journal of Pharmacology and Experimental Therapeutics 342, 619–630 (2012). 22700435 10.1124/jpet.112.192138 PMC3422529

[10] Pessoa J., Duarte A. I., Overcoming mitochondrial dysfunction in neurodegenerative diseases. Neural Regen Res 18, 1486 (2022). 10.4103/1673-5374.360279 PMC10075101 36571346

[11] Norat P., Soldozy S., Sokolowski J. D., Gorick C. M., Kumar J. S., Chae Y., Yağmurlu K., Prada F., Walker M., Levitt M. R., Price R. J., Tvrdik P., Kalani M. Y. S., Mitochondrial dysfunction in neurological disorders: Exploring mitochondrial transplantation. NPJ Regen Med 5, 22 (2020). 33298971 10.1038/s41536-020-00107-x PMC7683736

[12] Morton K. S., George A. J., Meyer J. N., Complex I superoxide anion production is necessary and sufficient for complex I inhibitor-induced dopaminergic neurodegeneration in Caenorhabditis elegans. Redox Biol 81, 103538 (2025). 39952197 10.1016/j.redox.2025.103538 PMC11875150

[13] González-Rodríguez P., Zampese E., Stout K. A., Guzman J. N., Ilijic E., Yang B., Tkatch T., Stavarache M. A., Wokosin D. L., Gao L., Kaplitt M. G., López-Barneo J., Schumacker P. T., Surmeier D. J., Disruption of mitochondrial complex I induces progressive parkinsonism. Nature 599, 650–656 (2021). 34732887 10.1038/s41586-021-04059-0 PMC9189968

[14] Markovich Z. R., Hartman J. H., Ryde I. T., Hershberger K. A., Joyce A. S., Ferguson P. L., Meyer J. N., Mild pentachlorophenol-mediated uncoupling of mitochondria depletes ATP but does not cause an oxidized redox state or dopaminergic neurodegeneration in Caenorhabditis elegans. Curr Res Toxicol 3, 100084 (2022). 35957653 10.1016/j.crtox.2022.100084 PMC9361317

[15] Delp J., Cediel-Ulloa A., Suciu I., Kranaster P., van Vugt-Lussenburg B. M., Munic Kos V., van der Stel W., Carta G., Bennekou S. H., Jennings P., van de Water B., Forsby A., Leist M., Neurotoxicity and underlying cellular changes of 21 mitochondrial respiratory chain inhibitors. Arch Toxicol 95, 591–615 (2021). 33512557 10.1007/s00204-020-02970-5 PMC7870626

[16] Meyer J. N., Leuthner T. C., Luz A. L., Mitochondrial fusion, fission, and mitochondrial toxicity. Toxicology 391, 42–53 (2017). 28789970 10.1016/j.tox.2017.07.019 PMC5681418

[17] Meyer D., Williams P. L., Toxicity testing of neurotoxic pesticides in Caenorhabditis elegans. J Toxicol Environ Health B Crit Rev 17, 284–306 (2014). 25205216 10.1080/10937404.2014.933722

[18] Sherer T. B., Betarbet R., Testa C. M., Seo B. B., Richardson J. R., Kim J. H., Miller G. W., Yagi T., Matsuno-Yagi A., Greenamyre J. T., Mechanism of Toxicity in Rotenone Models of Parkinson’s Disease. Journal of Neuroscience 23, 10756–10764 (2003). 14645467 10.1523/JNEUROSCI.23-34-10756.2003 PMC6740985

[19] Avila D., Helmcke K., Aschner M., The Caenorhabiditis elegans model as a reliable tool in neurotoxicology. Hum Exp Toxicol 31, 236–243 (2012). 21148196 10.1177/0960327110392084 PMC4917367

[20] Ma X., Jin M., Cai Y., Xia H., Long K., Liu J., Yu Q., Yuan J., Mitochondrial electron transport chain complex III is required for antimycin A to inhibit autophagy. Chem Biol 18, 1474–81 (2011). 22118681 10.1016/j.chembiol.2011.08.009 PMC3225892

[21] Luz A. L., Kassotis C. D., Stapleton H. M., Meyer J. N., The high-production volume fungicide pyraclostrobin induces triglyceride accumulation associated with mitochondrial dysfunction, and promotes adipocyte differentiation independent of PPARγ activation, in 3T3-L1 cells. Toxicology 393, 150–159 (2018). 29127035 10.1016/j.tox.2017.11.010 PMC5726929

[22] Inc. AAT Bioquest, EC50 Calculator. [Preprint] (2025).

[23] Maurer L. L., Philbert M. A., The mechanisms of neurotoxicity and the selective vulnerability of nervous system sites. Handb Clin Neurol 131, 61–70 (2015). 26563783 10.1016/B978-0-444-62627-1.00005-6

[24] Bijwadia S. R., Morton K., Meyer J. N., Quantifying Levels of Dopaminergic Neuron Morphological Alteration and Degeneration in Caenorhabditis elegans. J Vis Exp, doi: 10.3791/62894 (2021). PMC8815112 34866619

[25] Huayta J., Seay S., Laster J., Rivera N. A., Joyce A. S., Ferguson P. L., Hsu-Kim H., Meyer J. N., Assessment of developmental neurotoxicology-associated alterations in neuronal architecture and function using Caenorhabditis elegans. ALTEX, doi: 10.14573/altex.2501151 (2025). PMC12319415 40302409

[26] Mullin S., Schapira A. H. V., Pathogenic Mechanisms of Neurodegeneration in Parkinson Disease. Neurol Clin 33, 1–17 (2015). 25432720 10.1016/j.ncl.2014.09.010

[27] Rocha E. M., De Miranda B., Sanders L. H., Alpha-synuclein: Pathology, mitochondrial dysfunction and neuroinflammation in Parkinson’s disease. Neurobiol Dis 109, 249–257 (2018). 28400134 10.1016/j.nbd.2017.04.004

[28] Vozdek R., Pramstaller P. P., Hicks A. A., Functional Screening of Parkinson’s Disease Susceptibility Genes to Identify Novel Modulators of α-Synuclein Neurotoxicity in Caenorhabditis elegans. Front Aging Neurosci 14, 806000 (2022). 35572147 10.3389/fnagi.2022.806000 PMC9093606

[29] Bornhorst J., Chakraborty S., Meyer S., Lohren H., Brinkhaus S. G., Knight A. L., Caldwell K. A., Caldwell G. A., Karst U., Schwerdtle T., Bowman A., Aschner M., The effects of pdr1, djr1.1 and pink1 loss in manganese-induced toxicity and the role of α-synuclein in C. elegans. Metallomics 6, 476–90 (2014). 24452053 10.1039/c3mt00325f PMC3954834

[30] Nass R., Hall D. H., Miller D. M., Blakely R. D., Neurotoxin-induced degeneration of dopamine neurons in Caenorhabditis elegans. Proc Natl Acad Sci U S A 99, 3264–9 (2002). 11867711 10.1073/pnas.042497999 PMC122507

[31] Brand M. D., The sites and topology of mitochondrial superoxide production. Exp Gerontol 45, 466–472 (2010). 20064600 10.1016/j.exger.2010.01.003 PMC2879443

[32] Dröse S., Brandt U., Molecular mechanisms of superoxide production by the mitochondrial respiratory chain. Adv Exp Med Biol 748, 145–169 (2012). 22729857 10.1007/978-1-4614-3573-0_6

[33] Hanson G. T., Aggeler R., Oglesbee D., Cannon M., Capaldi R. A., Tsien R. Y., Remington S. J., Investigating mitochondrial redox potential with redox-sensitive green fluorescent protein indicators. J Biol Chem 279, 13044–53 (2004). 14722062 10.1074/jbc.M312846200

[34] Morton K. S., Hartman J. H., Heffernan N., Ryde I. T., Kenny-Ganzert I. W., Meng L., Sherwood D. R., Meyer J. N., Chronic high-sugar diet in adulthood protects Caenorhabditis elegans from 6-OHDA-induced dopaminergic neurodegeneration. BMC Biol 21 (2023). 10.1186/s12915-023-01733-9 PMC10636816 37950228

[35] Tantama M., Martínez-François J. R., Mongeon R., Yellen G., Imaging energy status in live cells with a fluorescent biosensor of the intracellular ATP-to-ADP ratio. Nat Commun 4, 2550 (2013). 24096541 10.1038/ncomms3550 PMC3852917

[36] An J. H., Vranas K., Lucke M., Inoue H., Hisamoto N., Matsumoto K., Blackwell T. K., Regulation of the Caenorhabditis elegans oxidative stress defense protein SKN-1 by glycogen synthase kinase-3. Proc Natl Acad Sci U S A 102, 16275 (2005). 16251270 10.1073/pnas.0508105102 PMC1283458

[37] Miranda-Vizuete A., Veal E. A., Caenorhabditis elegans as a model for understanding ROS function in physiology and disease. Redox Biol 11, 708 (2016). 28193593 10.1016/j.redox.2016.12.020 PMC5304259

[38] Gonzalez-Hunt C. P., Luz A. L., Ryde I. T., Turner E. A., Ilkayeva O. R., Bhatt D. P., Hirschey M. D., Meyer J. N., Multiple metabolic changes mediate the response of Caenorhabditis elegans to the complex I inhibitor rotenone. Toxicology 447, 152630 (2021). 33188857 10.1016/j.tox.2020.152630 PMC7750303

[39] Zuryn S., Kuang J., Tuck A., Ebert P. R., Mitochondrial dysfunction in Caenorhabditis elegans causes metabolic restructuring, but this is not linked to longevity. Mech Ageing Dev 131, 554–561 (2010). 20688098 10.1016/j.mad.2010.07.004

[40] Falk M. J., The pursuit of precision mitochondrial medicine: Harnessing preclinical cellular and animal models to optimize mitochondrial disease therapeutic discovery. J Inherit Metab Dis 44, 312–324 (2021). 33006762 10.1002/jimd.12319 PMC7994194

[41] Polyak E., Ostrovsky J., Peng M., Dingley S. D., Tsukikawa M., Kwon Y. J., McCormack S. E., Bennett M., Xiao R., Seiler C., Zhang Z., Falk M. J., N-acetylcysteine and vitamin E rescue animal longevity and cellular oxidative stress in pre-clinical models of mitochondrial complex I disease. Mol Genet Metab 123, 449–462 (2018). 29526616 10.1016/j.ymgme.2018.02.013 PMC5891356

[42] Guha S., Mathew N. D., Konkwo C., Ostrovsky J., Kwon Y. J., Polyak E., Seiler C., Bennett M., Xiao R., Zhang Z., Nakamaru-Ogiso E., Falk M. J., Combinatorial glucose, nicotinic acid and N-Acetylcysteine therapy has synergistic effect in preclinical C. elegans and zebrafish models of mitochondrial complex i disease. Hum Mol Genet 30, 536–551 (2021). 33640978 10.1093/hmg/ddab059 PMC8120136

[43] Orr A. L., Vargas L., Turk C. N., Baaten J. E., Matzen J. T., Dardov V. J., Attle S. J., Li J., Quackenbush D. C., Goncalves R. L. S., V Perevoshchikova I., Petrassi H. M., Meeusen S. L., Ainscow E. K., Brand M. D., Suppressors of superoxide production from mitochondrial complex III. Nat Chem Biol 11, 834–6 (2015). 26368590 10.1038/nchembio.1910 PMC4618194

[44] Wong H.-S., Dighe P. A., Mezera V., Monternier P.-A., Brand M. D., Production of superoxide and hydrogen peroxide from specific mitochondrial sites under different bioenergetic conditions. J Biol Chem 292, 16804–16809 (2017). 28842493 10.1074/jbc.R117.789271 PMC5641882

[45] Vanden Hoek T. L., Becker L. B., Shao Z., Li C., Schumacker P. T., Reactive oxygen species released from mitochondria during brief hypoxia induce preconditioning in cardiomyocytes. Journal of Biological Chemistry 273, 18092–18098 (1998). 9660766 10.1074/jbc.273.29.18092

[46] Young T. A., Cunningham C. C., Bailey S. M., Reactive oxygen species production by the mitochondrial respiratory chain in isolated rat hepatocytes and liver mitochondria: Studies using myxothiazol. Arch Biochem Biophys 405, 65–72 (2002). 12176058 10.1016/s0003-9861(02)00338-7

[47] Tahara E. B., Navarete F. D. T., Kowaltowski A. J., Tissue-, substrate-, and site-specific characteristics of mitochondrial reactive oxygen species generation. Free Radic Biol Med 46, 1283–97 (2009). 19245829 10.1016/j.freeradbiomed.2009.02.008

[48] Sun Y., Li T., Xie C., Zhang Y., Zhou K., Wang X., Blomgren K., Zhu C., Dichloroacetate treatment improves mitochondrial metabolism and reduces brain injury in neonatal mice. Oncotarget 7, 31708–31722 (2016). 27153546 10.18632/oncotarget.9150 PMC5077971

[49] Lavorato M., Nakamaru-Ogiso E., Mathew N. D., Herman E., Shah N., Haroon S., Xiao R., Seiler C., Falk M. J., Dichloroacetate improves mitochondrial function, physiology, and morphology in FBXL4 disease models. JCI Insight 7 (2022). 10.1172/jci.insight.156346 PMC9462489 35881484

[50] Broxton C. N., Kaur P., Lavorato M., Ganesh S., Xiao R., Mathew N. D., Nakamaru-Ogiso E., Anderson V. E., Falk M. J., Dichloroacetate and thiamine improve survival and mitochondrial stress in a C. elegans model of dihydrolipoamide dehydrogenase deficiency. JCI Insight 7 (2022). 10.1172/jci.insight.156222 PMC9714793 36278487

[51] Doonan R., McElwee J. J., Matthijssens F., Walker G. A., Houthoofd K., Back P., Matscheski A., Vanfleteren J. R., Gems D., Against the oxidative damage theory of aging: superoxide dismutases protect against oxidative stress but have little or no effect on life span in Caenorhabditis elegans. Genes Dev 22, 3236–41 (2008). 19056880 10.1101/gad.504808 PMC2600764

[52] Hunter T., Bannister W. H., Hunter G. J., Cloning, expression, and characterization of two manganese superoxide dismutases from Caenorhabditis elegans. Journal of Biological Chemistry 272, 28652–28659 (1997). 9353332 10.1074/jbc.272.45.28652

[53] Cabreiro F., Ackerman D., Doonan R., Araiz C., Back P., Papp D., Braeckman B. P., Gems D., Increased life span from overexpression of superoxide dismutase in Caenorhabditis elegans is not caused by decreased oxidative damage. Free Radic Biol Med 51, 1575–1582 (2011). 21839827 10.1016/j.freeradbiomed.2011.07.020 PMC3202636

[54] Keaney M., Matthijssens F., Sharpe M., Vanfleteren J., Gems D., Superoxide dismutase mimetics elevate superoxide dismutase activity in vivo but do not retard aging in the nematode Caenorhabditis elegans. Free Radic Biol Med 37, 239–50 (2004). 15203195 10.1016/j.freeradbiomed.2004.04.005

[55] Sampayo J. N., Olsen A., Lithgow G. J., Oxidative stress in Caenorhabditis elegans: protective effects of superoxide dismutase/catalase mimetics. Aging Cell 2, 319–26 (2003). 14677634 10.1046/j.1474-9728.2003.00063.x

[56] Onukwufor J. O., Farooqi M. A., Vodičková A., Koren S. A., Baldzizhar A., Berry B. J., Beutner G., Porter G. A., Belousov V., Grossfield A., Wojtovich A. P., A reversible mitochondrial complex I thiol switch mediates hypoxic avoidance behavior in C. elegans. Nat Commun 13, 2403 (2022). 35504873 10.1038/s41467-022-30169-y PMC9064984

[57] Nomura H., Athauda S. B. P., Wada H., Maruyama Y., Takahashi K., Inoue H., Identification and reverse genetic analysis of mitochondrial processing peptidase and the core protein of the cytochrome bc1 complex of Caenorhabditis elegans, a model parasitic nematode. J Biochem 139, 967–979 (2006). 16788047 10.1093/jb/mvj114

[58] Wojtovich A. P., Foster T. H., Optogenetic control of ROS production. Redox Biol 2, 368–376 (2014). 24563855 10.1016/j.redox.2014.01.019 PMC3926119

[59] Mandemakers W., Morais V. A., De Strooper B., A cell biological perspective on mitochondrial dysfunction in Parkinson disease and other neurodegenerative diseases. J Cell Sci 120, 1707–1716 (2007). 17502481 10.1242/jcs.03443

[60] Attene-Ramos M. S., Huang R., Michael S., Witt K. L., Richard A., Tice R. R., Simeonov A., Austin C. P., Xia M., Profiling of the Tox21 chemical collection for mitochondrial function to identify compounds that acutely decrease mitochondrial membrane potential. Environ Health Perspect 123, 49–56 (2015). 25302578 10.1289/ehp.1408642 PMC4286281

[61] Wills L. P., Beeson G. C., Hoover D. B., Schnellmann R. G., Beeson C. C., Assessment of ToxCast Phase II for Mitochondrial Liabilities Using a High-Throughput Respirometric Assay. Toxicol Sci 146, 226–234 (2015). 25926417 10.1093/toxsci/kfv085 PMC5009421

[62] Meyer J. N., Leung M. C. K., Rooney J. P., Sendoel A., Hengartner M. O., Kisby G. E., Bess A. S., Mitochondria as a target of environmental toxicants. Toxicol Sci 134, 1–17 (2013). 23629515 10.1093/toxsci/kft102 PMC3693132

[63] Kamp F., Exner N., Lutz A. K., Wender N., Hegermann J., Brunner B., Nuscher B., Bartels T., Giese A., Beyer K., Eimer S., Winklhofer K. F., Haass C., Inhibition of mitochondrial fusion by α-synuclein is rescued by PINK1, Parkin and DJ-1. EMBO J 29, 3571 (2010). 20842103 10.1038/emboj.2010.223 PMC2964170

[64] Hartman J. H., Gonzalez-Hunt C., Hall S. M., Ryde I. T., Caldwell K. A., Caldwell G. A., Meyer J. N., Genetic defects in mitochondrial dynamics in caenorhabditis elegans impact ultraviolet c radiation- and 6-hydroxydopamine-induced neurodegeneration. Int J Mol Sci 20, 3202 (2019). 31261893 10.3390/ijms20133202 PMC6651461

[65] Rehman K., Fatima F., Waheed I., Akash M. S. H., Prevalence of exposure of heavy metals and their impact on health consequences. J Cell Biochem 119, 157–184 (2018). 28643849 10.1002/jcb.26234

[66] Shan L., Heusinkveld H. J., Paul K. C., Hughes S., Darweesh S. K. L., Bloem B. R., Homberg J. R., Towards improved screening of toxins for Parkinson’s risk. npj Parkinson’s Disease 2023 9:1 9, 1–10 (2023). 10.1038/s41531-023-00615-9 PMC10730534 38114496

[67] Esser L., Xia D., Mitochondrial Cytochrome bc1 Complex as Validated Drug Target: A Structural Perspective. Trop Med Infect Dis 9 (2024). 10.3390/tropicalmed9020039 PMC10892539 38393128

[68] Boyd W. A., V Smith M., Freedman J. H., Caenorhabditis elegans as a model in developmental toxicology. Methods Mol Biol 889, 15–24 (2012). 22669657 10.1007/978-1-61779-867-2_3 PMC3513774

[69] Stiernagle T., Maintenance of C. elegans. WormBook, 1–11 (2006). 10.1895/wormbook.1.101.1 PMC4781397 18050451

[70] Avery L., Shtonda B. B., Food transport in the C. elegans pharynx. J Exp Biol 206, 2441–57 (2003). 12796460 10.1242/jeb.00433 PMC3951750

[71] Moore B. T., Jordan J. M., Baugh L. R., WormSizer: high-throughput analysis of nematode size and shape. PLoS One 8, e57142 (2013). 23451165 10.1371/journal.pone.0057142 PMC3579787

[72] Morton K. S., Wahl A. K., Meyer J. N., The effect of common paralytic agents used for fluorescence imaging on redox tone and ATP levels in Caenorhabditis elegans. PLoS One 19 (2024). 10.1371/journal.pone.0292415 PMC11051652 38669260

