# Supplementary material for: Inhibition of Mitochondrial Complex III Causes Dopaminergic Neurodegeneration by Redox Stress in Caenorhabditis elegans
Source: bioRxiv. 2025 Oct 23:2025.10.21.683798. Preprint. [Version 1] doi: 10.1101/2025.10.21.683798 (PMC12633311; doi:10.1101/2025.10.21.683798)
Supplement: Supplement 1 [file media-1.pdf]

Supplementary materials for:

Inhibition of Mitochondrial Complex III Causes Dopaminergic Neurodegeneration by Redox Stress in *Caenorhabditis elegans*

Javier Huayta, Joel N. Meyer[*]

[*] Corresponding author joel.meyer@duke.edu

This PDF file includes:

Figs. S1 to S7

Tables S1 to S2

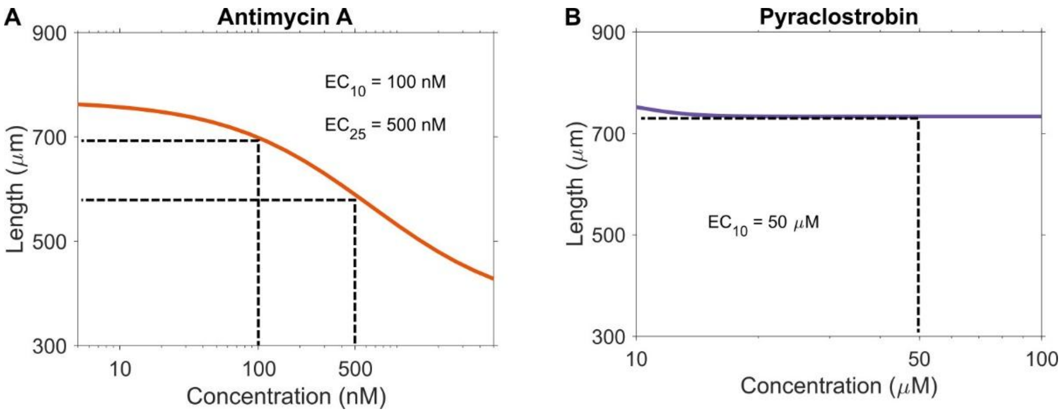

Fig. S1: Dose response curves. Length-based response curves for (**A**) antimycin A and (**B**) pyraclostrobin. EC_{10} = 100 nM and EC_{25} = 500 nM for antimycin A, EC_{10} = 50 μM for pyraclostrobin.

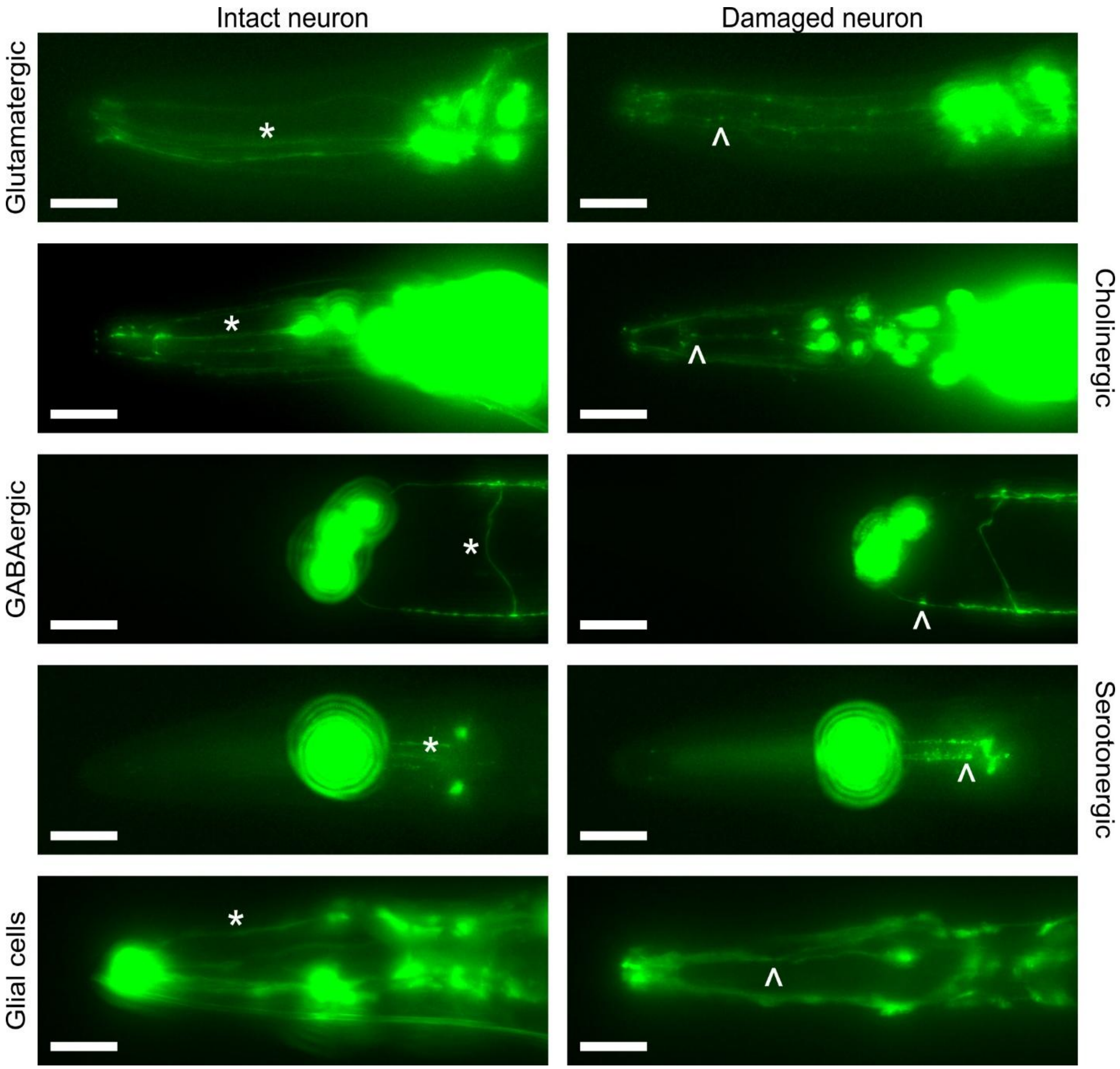

Fig. S2. Example images of neuronal damage. Representative images of intact neurites (marked with a "*" symbol) and damaged neurites (marked with a "^" symbol) showing blebs and breaks. From top to bottom: glutamatergic, cholinergic, GABAergic, and serotonergic neurons, and glial cells located in the head of *C. elegans*. Scale bars 20 µm.

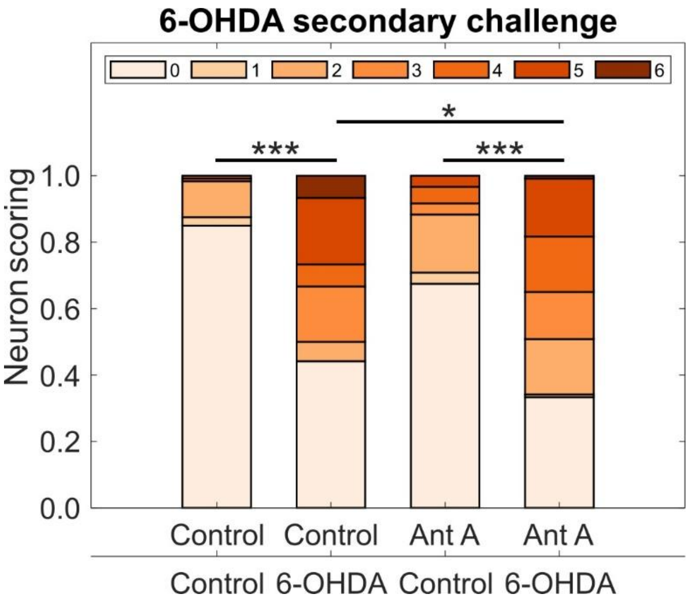

Fig. S3. Neurodegeneration after 6-OHDA secondary challenge. Neuronal damage scoring distribution for cephalic dopaminergic neurons after one hour challenge with 50 mM 6-OHDA following developmental exposure to 500 nM antimycin A. $N = 3$ biological replicates, $n = 40$ dendrites per treatment per replicate, chi-square test with Bonferroni post-hoc test, *$P < 0.05$, ***$P < 0.001$.

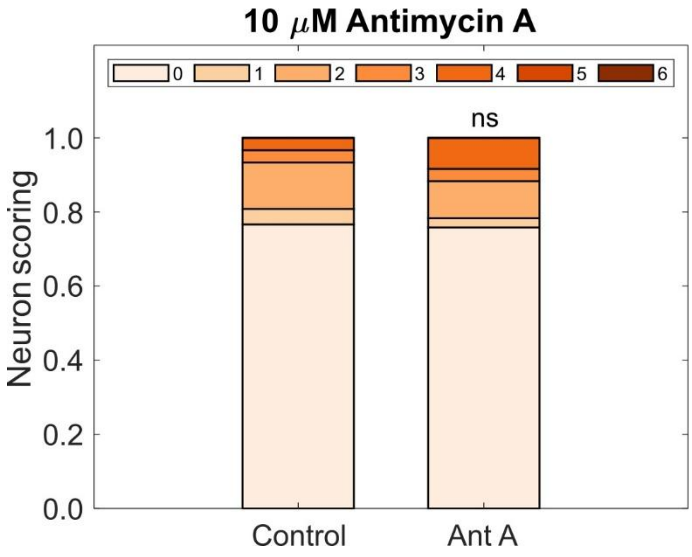

Fig. S4. Acute exposure to antimycin A does not cause dopaminergic neurodegeneration. Neuronal damage scoring distribution for cephalic dopaminergic neurons after two and a half hours exposure to 10 μM antimycin A at the L4 larval stage. $N = 3$ biological replicates, $n = 40$ dendrites per treatment per replicate, chi-square test with Bonferroni post-hoc test, ns $P > 0.05$.

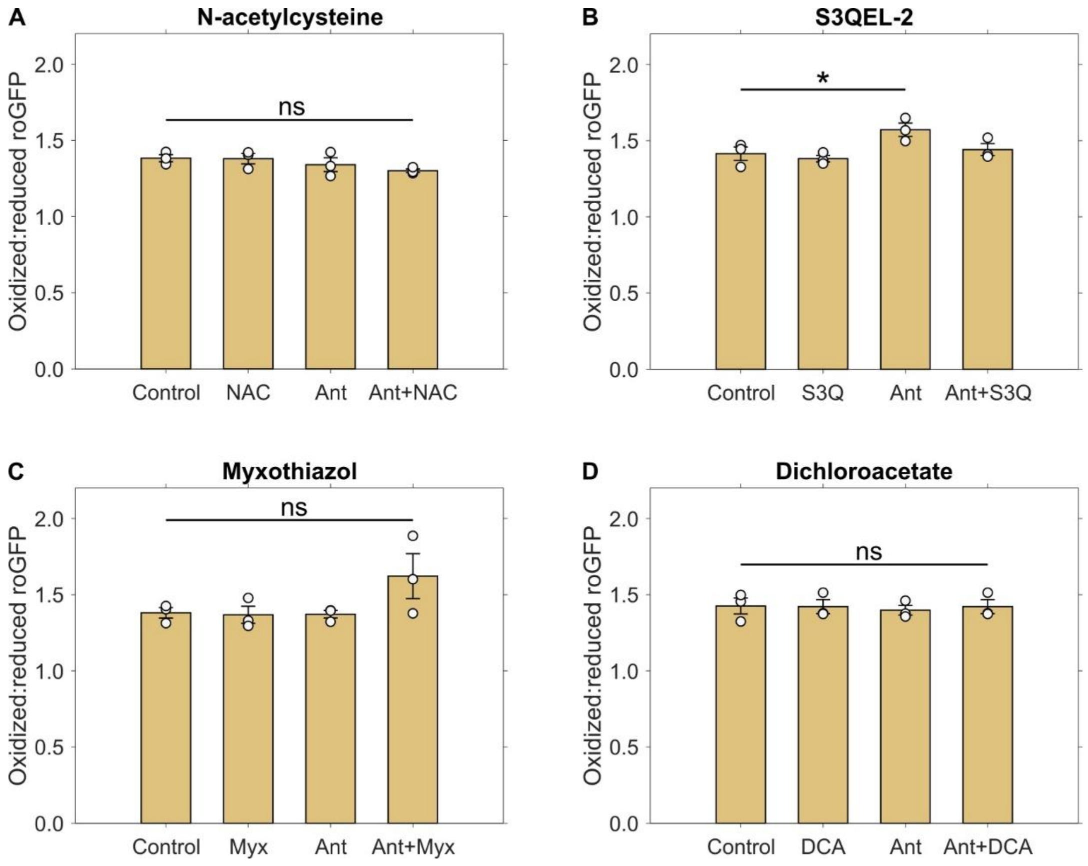

Fig. S5. Dopaminergic redox state after chemical rescue. Oxidized to reduced roGFP ratio in cephalic dopaminergic neurons of *C. elegans* developmentally exposed to 500 nM antimycin A with (**A**) 2.5 mM N-acetylcysteine, (**B**) 100 μM S3QEL-2, (**C**) 10 μM myxothiazol, and (**D**) 25 mM dichloroacetate. $N = 3$ biological replicates, $n = 40$ neurons per treatment per replicate, one-way ANOVA test with Dunnett's post-hoc test, ns $P > 0.05$, *$P < 0.05$.

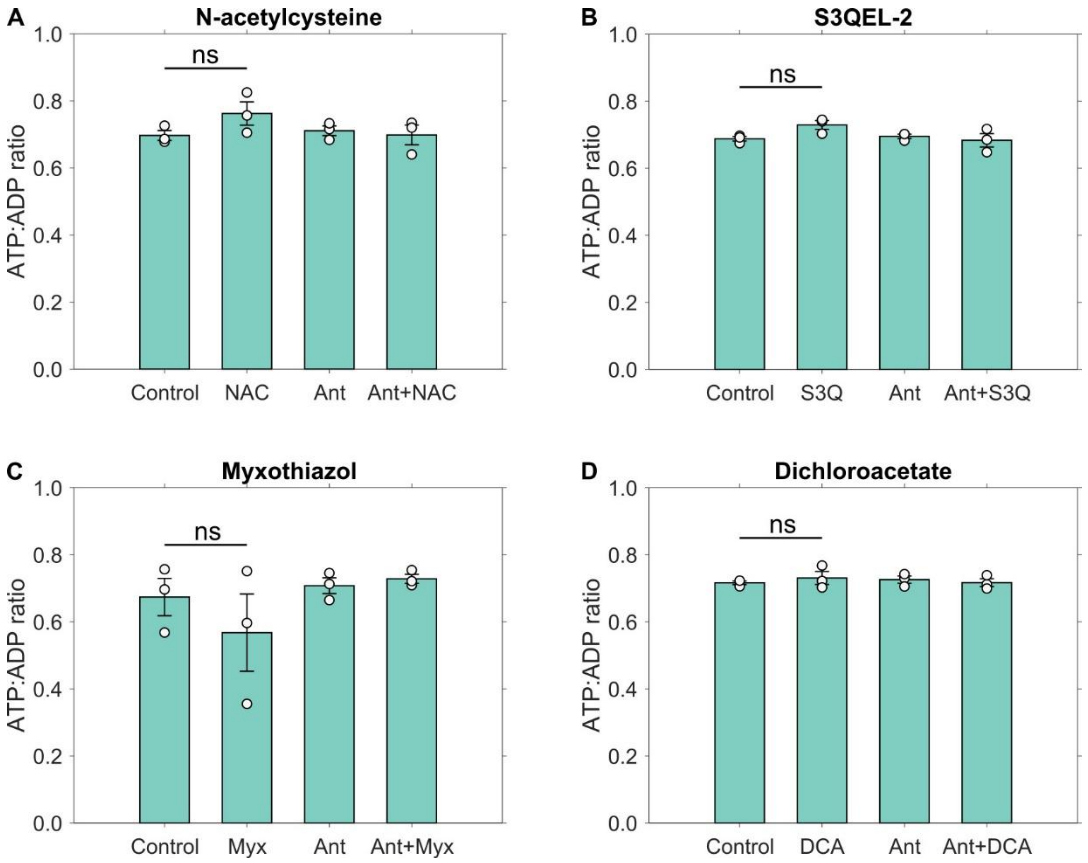

Fig. S6. Dopaminergic ATP levels after chemical rescue. ATP to ADP ratio measured using the PercevalHR reporter in cephalic dopaminergic neurons of *C. elegans* developmentally exposed to 500 nM antimycin A with (**A**) 2.5 mM N-acetylcysteine, (**B**) 100 µM S3QEL-2, (**C**) 10 µM myxothiazol, and (**D**) 25 mM dichloroacetate. $N = 3$ biological replicates, $n = 40$ neurons per treatment per replicate, one-way ANOVA test with Dunnett's post-hoc test, ns $P > 0.05$.

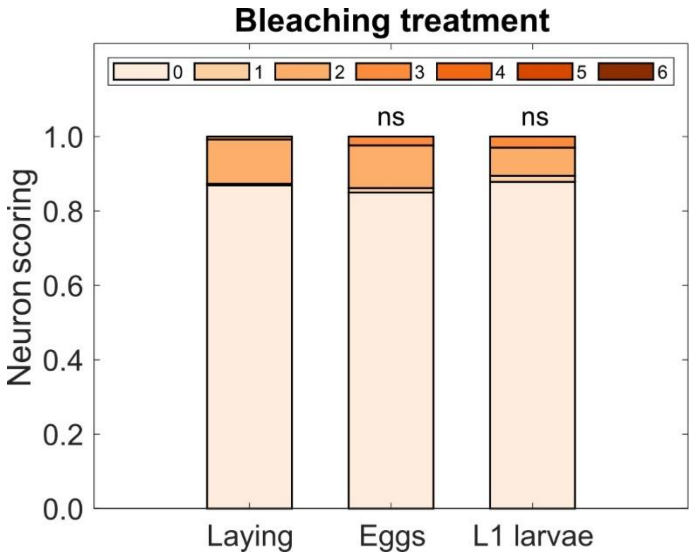

Fig. S7. Neurodegeneration after bleaching treatment. Neuronal damage scoring distribution for cephalic dopaminergic neurons measure at the L4 larval stage of *C. elegans* populations age-synchronized by letting adults lay eggs, egg recovery from gravid adults by bleaching treatment, or gravid adult bleaching treatment followed with overnight egg hatching to produce L1 larval stage worms. $N = 3$ biological replicates, $n = 80$ dendrites per treatment per replicate, chi-square test with Bonferroni post-hoc test, ns $P > 0.05$.

Table S1: List of *C. elegans* strains.

**Strain name**	**Genotype**	**Description**
BY200	*vtIs1*[p*dat-1*::GFP]	Dopaminergic reporter
DA1240	*adIs1240*[*eat-4*::sGFP+*lin-15*(+)]	Glutamatergic reporter
LX929	*vsIs48*[*unc-17*::GFP]	Cholinergic reporter
CZ1632	*juIs76*[*unc-25*p::GFP+*lin-15*(+)]	GABAergic reporter
GR1366	*mgIs42*[*tph-1*::GFP+*rol-6*(su1006)]	Serotonergic reporter
VT1485	*maIs188*[*mir-228*p::GFP+*unc-119*(+)	Glial cell reporter
ERS1	*eraIs1*[*dat-1p*::mCherry, *dat-1p*::hSNCA::Venus]	Dopaminergic α-synuclein
ERS44	*eraIs1*[*dat-1p*::mCherry, *dat-1p*::hSNCA::Venus]; *pdr-1*(gk448)	Dopaminergic α-synuclein, *pdr-1* mutant
ERS49	*eraIs1*[*dat-1p*::mCherry, *dat-1p*::hSNCA::Venus]; *pink-1(tm1779)*	Dopaminergic α-synuclein, *pink-1* mutant
UA226	*pink-1*(tm1779); *vtIs1*[p*dat-1*::GFP]	Dopaminergic reporter, *pink-1* mutant
UA227	*pdr-1*(tm598); *vtIs1*[p*dat-1*::GFP]	Dopaminergic reporter, *pdr-1* mutant
PHX2867	p*dat-1*::MLS::roGFP	Dopaminergic roGFP reporter
PHX2923	p*dat-1*::PercevalHR	Dopaminergic PercevalHR reporter
GA184	*sod-2*(gk257) I	*sod-2* mutant
GA186	*sod-3*(tm760) X	*sod-3* mutant
GA805	*wuIs156*[*sod-2*(genomic) + *rol-6*(su1006)	*sod-2* over expressor
APW125	*jbm21*[*ucr2.3*::link::SuperNova] III	Complex III SuperNova
BY200 x GA184	*vtIs1*[p*dat-1*::GFP]; *sod-2*(gk257) I	Dopaminergic reporter, *sod-2* mutant
BY200 x GA186	*vtIs1*[p*dat-1*::GFP]; *sod-3*(tm760) X	Dopaminergic reporter, *sod-3* mutant
BY200 x GA805	*vtIs1*[p*dat-1*::GFP]; *wuIs156*[*sod-2*(genomic) + *rol-6*(su1006)	Dopaminergic reporter, *sod-2* over expressor
BY200 x APW125	*vtIs1*[p*dat-1*::GFP]; *jbm21*[*ucr2.3*::link::SuperNova] III	Dopaminergic reporter, complex III SuperNova

Table S2: Primers used for PCR genotyping of *C. elegans* crosses

**Strain 1**	**Strain 2**	**Forward primer**	**Reverse primer**
BY200	GA184	CAAGTCCAGTTGTTGCCTCA	CAAAAACCACTTTGCTCGGT
BY200	GA186	TGTTTACTTTGTTCTCGTGGGTT	CCTTCCAAATAGCATGGACATAG
BY200	APW125	AGTCGAGATTAGCCCTTGGT	AAGCTCCTCTCTGTCTTGGC
